# Lamination of microfibrous PLGA fabric by electrospinning a layer of collagen-hydroxyapatite composite nanofibers for bone tissue engineering

**DOI:** 10.1186/s40824-017-0097-3

**Published:** 2017-06-13

**Authors:** Gi-Wan Kwon, Kailash Chandra Gupta, Kyung-Hye Jung, Inn-Kyu Kang

**Affiliations:** 1 Department of Polymer Science and Engineering, Kyungpook National University, Daegu, 702-701 South Korea; 2 Polymer Research Laboratory, Department of Chemistry, I. I. T. Roorkee, Roorkee, 247 667 India; 3 Department of Advanced Materials and Chemical Engineering,Catholic University of Daegu, Kyungsan, South Korea

**Keywords:** Collagen, PLGA, Polydopamine, Hydroxyapatite nanorods and Scaffolds

## Abstract

**Background:**

To mimic the muscle inspired cells adhesion through proteins secretion, the lamination of collagen–hydroxyapatite nanorod (nHA) composite nanofibers has been carried out successfully on polydopamine (PDA)-coated microfibrous polylactide-co-glycolide (PLGA) fabrics. The lamination of collagen-hydroxyapatite composite nanofibers on polydopamine-coated microfibrous PLGA fabrics was carried through electrospinning the solution of collagen containing L-glutamic acid-grafted hydroxyapatite nanorods (nHA-GA) at a flow rate of 1.5 mL/h and an applied voltage of 15 kV.

**Results:**

In comparison to pristine PLGA, dopamine-coated PLGA and collagen-hydroxyapatite composite nanofiber lamination has produced more wettable surfaces and surface wettability is found to higher with dopamine-coated PLGA fabrics then pristine PLGA. The SEM micrographs have clearly indicated that the lamination of polydopamine-coated PLGA fabric with collagen-hydroxyapatite composite nanofibers has shown increased adhesion of MC3T3E1 cells in comparison to pristine PLGA fabrics.

**Conclusion:**

The results of these studies have clearly demonstrated that collagen-nHA composites fibers may be used to create bioactive 3D scaffolds using PLGA as an architectural support agent.

## Background

The structures and properties of scaffold play significant role in tissue engineering, therefore, various techniques have been used frequently to design scaffolds using biocompatible materials of different structures and properties [1–3]. Amongst the various techniques of fabrication of scaffolds, the technique of electrospinning is found to be versatile and acceptable over the globe [4]. It is able to form continuous and uniform size fibers ranging from micro- to nano sized diameter [5] for various applications ranging from tissue engineering to the fabrication of drug delivery devices [6–8]. The scaffolds need to be compatible with neighboring tissues and able to provide sufficient sites for cells attachment. To fabricate bioactive surfaces with improved affinity for attachment of mesenchymal cell, the surface modifications have been made earlier either by carrying out chemical reaction with bioactive material or by simply coating a bioactive material [9–11]. To enhance the attachment of osteoblasts and their osseointegration on scaffolds, various bioactive materials such as; hydroxyapatite (HA) [12, 13], tricalcium phosphate (TCP) [14] and strontium containing hydroxyapatite have been used in combination with different polymeric materials [15]. The addition of ceramics in general has promoted cellular infiltration and differentiation but HA and TCP also helped in mineralization. The collagen in combination with bone morphogenetic protein-2 (rhBMP-2) has shown increasing effect on cells adhesion and differentiation on the scaffolds, which are fabricated using bio-inert materials such as polyetheretherketone (PEEK) [16]. In comparison to microfibrous scaffolds, the nanofibrous scaffolds seem to be highly bioactive due to having high surface to mass ratio and 3D nanostructures, which play significant role in cells adhesion, proliferation and differentiation in tissue engineering [17, 18]. We have used biodegradable poly(lactide-co-glycolide) (PLGA) in fabrication of scaffolds for tissue engineering using single [19, 20] and dual electrospining technique [21].

The poly(lactide-co-glycolide) is approved by FDA (USA) and often used in preference to pure PLLA, PLA, and PGA because its degradation rate is easily controlled by varying the ratio of glycolides to lactides segments in PLGA copolymer backbone. The structure and property of collagen Type I was found to be suitable and biocompatible for fabrication of scaffolds for tissue engineering [22, 23]. Collagen Type I is found to show significantly high cells attachment and penetration in comparison to scaffolds fabricated using PLGA or other materials. The scaffolds fabricated using a blend of synthetic polymers and collagen has shown high cells recognition in comparison to scaffolds fabricated taking synthetic polymers [24]. The coating of collagen-hydroxyapatite composite fibers on scaffolds fabricated with poly(lactide-co-glycolide)/β-tricalciumphosphate composites, has shown a significant improvement in alkaline phosphatage activity (ALP) in tissue engineering [25]. These studies have provided sufficient impetus to laminate electrospun microfibrous PLGA fabrics with collagen-hydroxyapatite composite nanofibers to obtain scaffolds with enhanced cells attachment and penetration. The microfibrous PLGA fabrics have played a significant role in providing mechanical strength and structural support to electrospun active layer of collagen-hydroxyapatite composites nanofibers, which induced cells attachment, proliferation, and differentiation. The microfibrous PLGA fabric laminated with collagen-hydroxyapatite composite nanofibers was characterized for surface wetting properties and morphology by contact angle measurements and recording SEM images of the scaffolds. The cell seeding experiments have confirmed that ionically bound collagen is found to be more bioactive than its bindings with weak van der Waal’s physical forces.

## Methods

### Chemicals and methodology

Poly(lactide-co-glycolide) (PLGA) with lactide to glycolide ratio 85:15 (MW, 240,000 Da), dopamine hydrochloride (DA) (MW, 89.64 g mol^−1^), L-glutamic acid (GA), tris(hydroxymethyl) aminomethane (Tris) buffer solution (pH 8.5), N-(3-dimethylaminopropyl)-N′-ethylcarbodiimide (EDC), N-hydroxysuccinimide (NHS), sodium dodecyl sulfate (SDS) (Mw, 288.38 g mol^−1^), and 3-(4,5-dimethylazol-2-yl)-2,5-diphenyl-2H-tetrazolium bromide (MTT) assay were purchased from Sigma-Aldrich Chemical Company, USA. Collagen Type I was purchased from Bioland Company, Korea. The hydroxyapatite nanorods (nHA) were synthesized as per details as given in our previous communication [20]. The mouse pre-osteoblast cells (MC3T3-E1) were purchased from Korea cells bank (Seoul, South Korea) and stored in liquid nitrogen before carrying out cells seeding experiments. The 10 × 10^−3^ mmol phosphatae buffer saline (PBS) solution (pH 7.4) containing 87 × 10^−3^ mmol Na_2_HPO_4_, 14 × 10^−3^ mmol KH_2_PO_4_, 131 × 10^−3^ mmol NaCl and 27 × 10^−3^ mmol KCl was purchased from Sigma-Aldrich Chemical Company, USA. The osteoblastic MC3T3-E1 cells were cultured in α-minimum essential medium (α-MEM) (Gibco BRL, Grand Island, NY, USA) supplemented with 10% fetal bovine serum (FBS; Gibco), 1.0% penicillin G-streptomycin at 37 °C under 5% CO_2_ atmosphere. The culture medium was changed every other day. The amount of self-polymerized dopamine on nonwoven microfibrous PLGA was determined by spectrometric analysis of unpolymerized dopamine in solution and washings at 350 nm. All other chemicals and solvents used in experimental work were of high purity reagents and purchased from Sigma-Aldrich Chemical Company, USA.

### Electrospinning of microfibrous PLGA fabrics

**Fig. 1:**
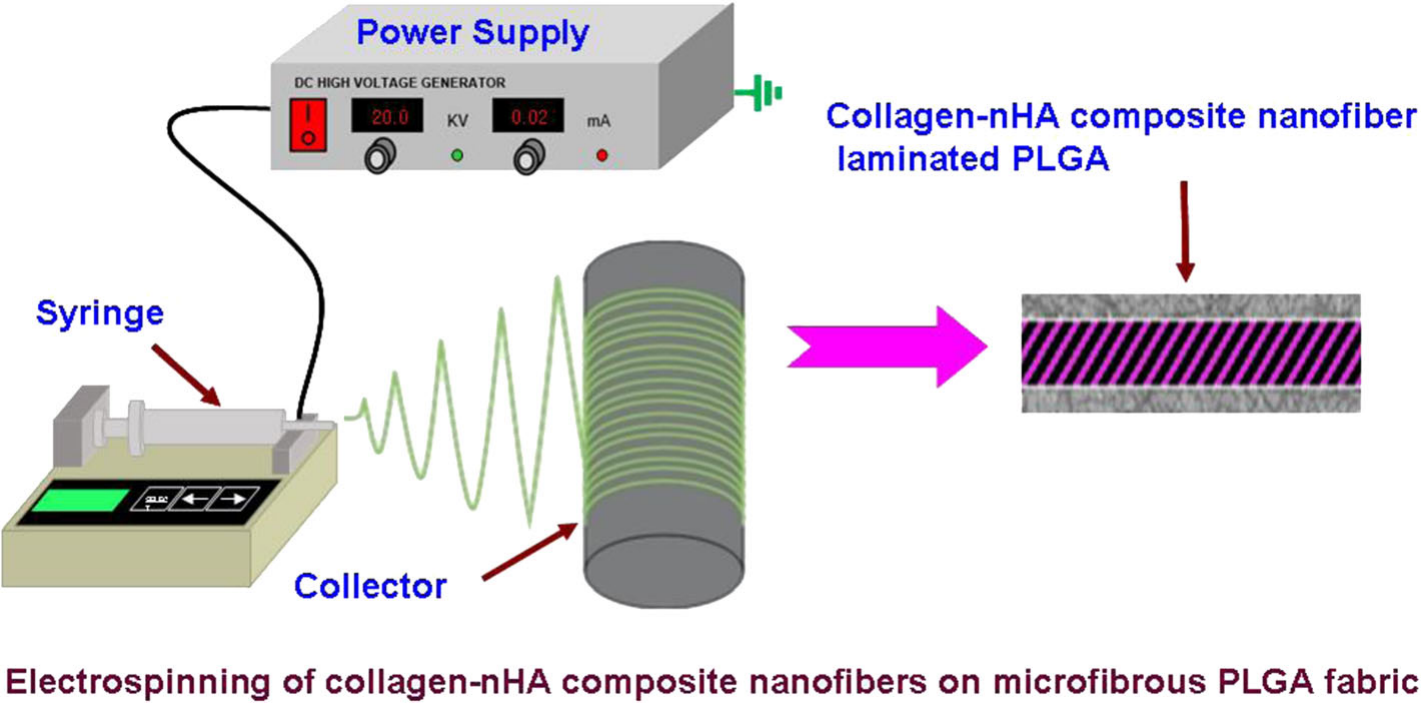
Electrospinning setup for spinning of micro and nanofibers

A 25 wt% solution of PLGA in a binary mixture of tetrahydrofuran and dimethyl formamide (3:1) was used to electrospun microfibrous PLGA fabrics. The solution of PLGA was used to electrospun microfibrous fabrics at a flow rate of 1.5 mL/h using 10 mL syringe fitted with 20G needle. The needle to collector distance was kept 15 cm. The microfibrous PLGA fabrics were electrospun by varying voltage 12 kV to 18 kV using a high-voltage direct-current power supply to optimize the voltage for electrospinning of PLGA fabrics. On application of voltage between needle and collector, the solution droplet was forced to leave the needle in the form of ultra fine fibers, which were deposited on collector (Fig. 1). The non-woven fabric was detached from collector after reaching an appropriate thickness and placed in vacuum for the evaporation of residual solvent. The prepared microfibrous PLGA fabrics after drying were subsequently used to laminate with collagen-hydroxyapatite composite nanofibers by electrospinning a solution of collagen containing 5 wt% L-glutamic acid modified-hydroxyapatite nanorods (nHA-GA).

### Preparation of polydopamine-coated microfibrous PLGA fabrics

**Fig. 2:**
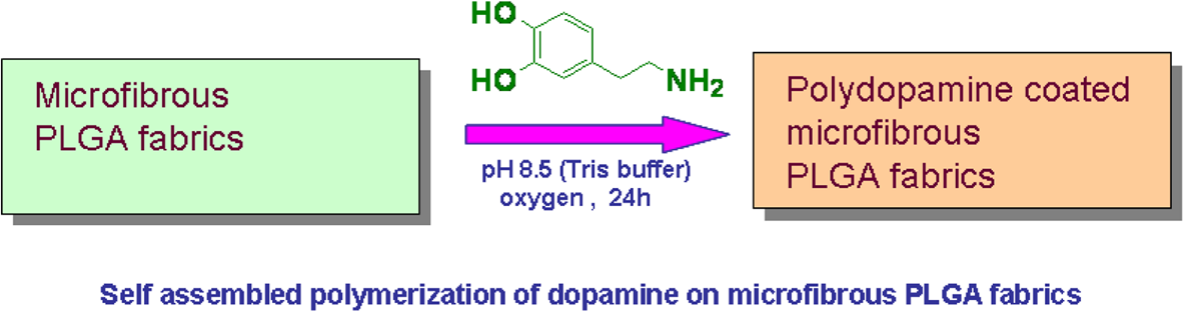
Coating of polydopamine on microfibrous PLGA fabrics

For covalent immobilization of collagen-hydroxyapatite composite nanofibers, the samples of microfibrous PLGA fabrics were coated with polydopamine to ensure covalent binding of collagen-hydroxyapatite composite nanofibers on PLGA fabrics. To carry out surface modifications of microfibrous PLGA fabrics with self assembled polydopamine, the samples of microfibrous PLGA fabrics were immerged in a alkaline Tris buffer solution (pH 8.5) of dopamine (10 mg/mL) and kept for about 24 h (Fig. 2). During this period, the samples of microfibrous PLGA fabric were coated with the layers of self assembled polydopamine (pDA), which helped in ionic adhesion of collagen-hydroxyapatite composites nanofibers (Col-nHA). The polydopamine-modified samples of microfibrous PLGA fabrics were washed with phosphate buffer saline solution (PBS) and subsequently used for lamination with the layers of collagen-hydroxyapatite composites nanofibers by electrospinning.

### Surface modification of hydroxyapatite nanorods (n-HA)

**Fig. 3:**
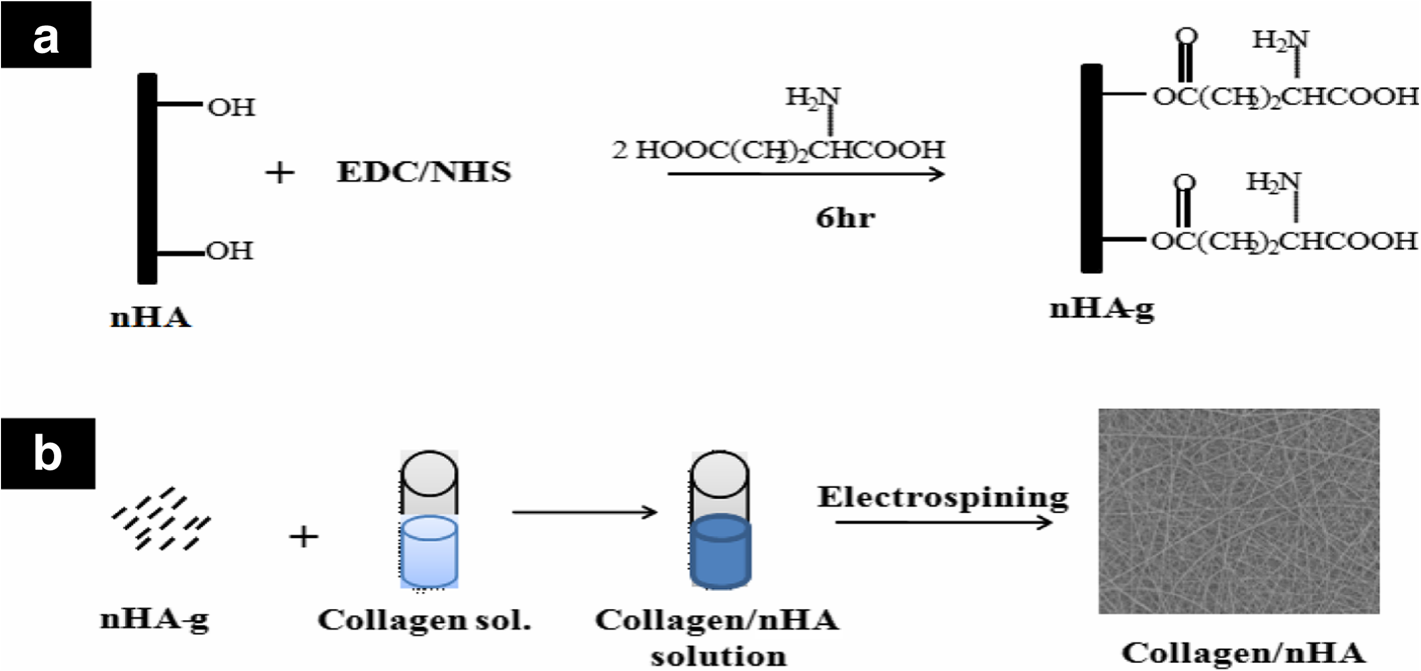
Grafting of glutamic acid on hydroxyapatite nanorods

To ensure homogeneous distribution of hydroxyapatite nanorods (nHA) in solution of collagen, the surface of hydroxyapatite nanorods (nHA) was modified by L-glutamic acid grafting as described in our previous communications [20, 21]. Briefly, the terminals carboxylic acid groups (COOH) of L-glutamic acid were activated by keeping in a mixture of water-soluble carbodiimide (1-ethyl-3-(3-dimethylaminopropyl) carbodiimide hydrochloride) (0.5 g, 0.25 wt%) and *N*-hydroxysuccinimide (0.5 g, 0.25 wt%) for about 6 h under constant stirring. After stirring for about 6 h, the L-glutamic acid-grafted nHA were centrifuged and dried after washing with deionized water (Fig. 3a). The L-glutamic-acid grafted nHA was mixed with collagen to electrospin nanofibers on microfibrous PLGA fibers (Fig. 3b).

### Electrospinning of collagen-hydroxyapatite composite nanofibers on dopamine-modified microfibrous PLGA fabrics

To laminate the samples of microfibrous PLGA fabrics with collagen-hydroxyapatite composite nanofibers, the self assembled polydopamine-modified microfibrous PLGA fabrics wrapped on a metal collector was used during electrospining. To laminate polydopamine-coated microfibrous PLGA fabrics with collagen-hydroxyapatite nanofibers, the solution of collagen (5 mg/mL) in 0.1 M carbonate-bicarbonate buffer (pH 9.5) containing homogeneously distributed L-glutamic acid-modified nHA (5.0 wt%, 50 mg/mL) was used. The solution of collagen and hydroxyapatite nanorods was electrospun at a flow rate of 1.5 mL/h and constant tip to collector distance (15 cm) and applied voltage (15 kV) (Fig. 1). After electrospinning, the solution of collagen-hydroxyapatite for about 6 h, the samples of collagen-hydroxyapatite nanofibers-coated PLGA fabrics were vacuum dried and then characterized for their surface wettability and morphology by contact angle measurements and recording their SEM micrographs. In order to determine the effect of concentration of hydroxyapatite nanorods on cells adhesion and osteogenic properties of collagen-hydroxyapatite composite nanofibers-modified microfibrous PLGA fabrics, the solution of collagen having different amount of hydroxyapatite nanorods was also used for electrospinning the layers of collagen-hydroxyapatite composite nanofibers on microfibrous PLGA fabrics. The amount of hydroxyapatite nanorods in solution of collagen was varied from 3.8-5.0 wt% (38-50 mg/mL). The electrospinning of collagen-nHA nanocomposite fibers was also carried out on PLGA fabrics without dopamine.

### Characterization of collagen-hydroxyapatite composite nanofibers-laminated microfibrous PLGA fabrics

The surface morphology of microfibrous PLGA fabric laminated with collagen-hydroxyapatite composite nanofibers is determined by recording SEM micrographs (FE-SEM, 400 Hitachi, Tokyo, Japan). The samples were fixed to SEM holder using double adhesive carbon tape and then sputter-coated with platinum. The platinum-coated samples were then examined by FE-SEM under high vacuum. The surface wettability of microfibrous PLGA fabrics laminated with collagen-hydroxyapatite composite nanofibers was evaluated by contact angle (θ) measurements, which were carried out by sessile drop method (Kruss contact angle equipment model DS10) and using deionized water, diiodomethane and formamide as reference solvents. The contact angle (θ) was reported as a mean of three measurements.

### Evaluation of cell attachment and proliferation properties of ionically bound collagen-hydroxyapatite nanofibrous composite layer

To determine the effect of ionically bound collagen on attachment of cells, the samples of microfibrous PLGA fabrics laminated with collagen-hydroxyapatite composite nanofibers were used to evaluate the adhesion of MC3T3E3 cells after seeding MC3T3-E1 cells (5 × 10^4^ cells/mL per sample) in a α-minimum essential medium supplemented with 10% fetal bovine serum and 1% penicillin/streptomycin. The MC3T3-E1 cells were incubated in a humidified atmosphere at 37 °C in presence of 5% CO_2_ for 12 h and 24 h. The cell seeding experiments were also repeated using samples of microfibrous PLGA, polydopamine coated microfibrous PLGA (PLGA-D) and microfibrous PLGA laminated with collagen-hydroxyapatite composite nanofibers (PLGA-Col/nHA). The results of cells attachment of these fabrics were compared with polydopamine-coated microfibrous PLGA fabric laminated with collagen-hydroxyapatite composite nanofibers (PLGA-D-Col/nHA). To confirm the cells attachment on fabrics, the SEM micrographs of cells seeded fabrics were recorded after fixing cells with 2.5% glutaraldehyde for 20 min. Finally scaffolds were dehydrated with critical point drier (EMS 850 Critical Point Dryer, Hatfield, PA, USA) and stored after drying to record their FE-SEM (400-Hitachi, Tokyo, Japan) micrographs.

The microfibrous PLGA, polydopamine coated microfibrous PLGA (PLGA-D) and microfibrous PLGA laminated with collagen-hydroxyapatite composite nanofibers (PLGA-Col/nHA) have been evaluated for proliferation by seeding MC3T3-E1 cells for 3 days at a cell density of 3 × 10^4^ cells/mL in 4-well plate and then applying MTT assay. The proliferation of MC3T3-E1 cells was monitored by adding MTT solution (50 μL, 5 mg/mL in PBS) to each well and incubating in a humidified atmosphere containing 5% CO_2_ at 37 °C. After 4 h, the medium was removed and converted dye was dissolved in acidic isopropanol (0.04 N HCl-isopropanol) by keeping solution for 30 min in dark at 25 °C. Finally, 100 μL solution of each sample was transferred to a 96-well plate and absorbance of converted dye was recorded using ultraviolet light at 570 nm using kinetic microplate reader (ELx800, Bio-Tek Instruments, Inc., Highland Park, VT, USA).

## Results and discussion

The surface properties of scaffolds play significant role in controlling cells adhesion, proliferation and their differentiation; hence, designing of scaffolds with desired functionality and surface area is potentially useful in tissue engineering. In comparison to PLGA, the collagen is more useful for attachment of cells but due to lack of sufficient mechanical strength its application in fabrication of scaffolds is limited. In order to utilize the various properties of collagen Type I in bone tissue engineering, efforts have been made to fabricate 3D scaffolds by immobilizing an active layer of collagen-hydroxyapatite composite nanofibers on a biocompatible microfibrous support of PLGA. This arrangement has provided ample opportunities to utilize the functional properties of collagen and its high surface area to enhance MC3T3-E1 cells attachment and their proliferation. The microporous PLGA support has controlled the mechanical strength of the composite scaffolds and facilitated the formation and penetration of microvilli for the attachment of cells at the surfaces of the scaffolds. It is also evident that ionically immobilized collagen is more bioactive toward cells adhesion, proliferation and early state osteogenic differentiation of preosteogenic cells [26–30]. To facilitate the ionic immobilization of collagen, the microfibrous PLGA fabrics were coated with polydopamine (3,4-dihydroxy-L-phenylalanine), which is kwon to have strong covalent and non-covalent interactions with collagen and other biomolecules containing amine and thiol groups [31–35]. To control the osteogenic properties of collagen-laminated scaffolds of PLGA for MC3T3-E1 cells, the collagen nanofibers containing L-glutamic acid-grafted hydroxyapatite nanorods (nHA-GA) were electrospun on microfibrous PLGA fabrics, which were with and without dopamine. The discussion of the results as below has provided significant insights to understand the role of various factors that contributed toward cells adherence to collagen-laminated PLGA fabrics.

### Electropinning of microfibrous PLGA

**Fig. 4:**
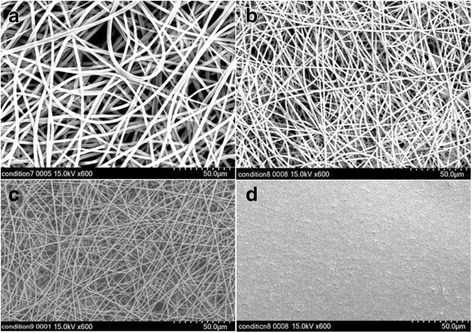
SEM micrographs of microfibrous PLGA fabrics electrospun at (**a**) 12 kV, (**b**) 15 kV, and (**c**) 18 kV at a flow rate of 1.5 mL/h and tip to collector distance of 15 cm and (**d**) dopamine-coated (24 h) microfibrous PLGA fabrics electrospun at 15 kV

To fabricate a microfibrous PLGA fabrics with suitable fiber size and porosity, a 25 wt% solution of PLGA in binary mixture of THF and DMF (3:1) was electrospun using a 10 mL syringe fitted with 20G needle and varying voltage from 12 to 18 kV at a flow rate of 1.5 mL/h at constant tip to collector distance of 15 cm (Fig. 4). The resultant PLGA microfibrous fabrics were analyzed by recording their SEM micrographs (Fig. 4, Table 1). The observation of SEM micrographs (Fig. 4) has made it clear that on increasing the applied voltage from 12 kV to 18 kV between needle and the grounded collector, the fiber diameter has shown a decreasing trend from 5.2 μm to 3.4 μm. This variation in fiber diameter is due to the increase in force on Taylor cone formed at the tip of the needle.

**Table 1:** Electrospinning parameters of microfibrous PLGA fabrics

Polymer/	Solvent	Voltage	TCD distance	Flow rate	Syringe	Fiber diameter
PLGA 25 wt%	THF:DMF 3:1	12 kV	15 cm	1.5 mL/h	20G	5.2 ± 0.014 μm
PLGA 25 wt%	THF:DMF 3:1	15 kV	15 cm	1.5 mL/h	20G	3.6 ± 0.012 μm
PLGA 25 wt%	THF:DMF 3:1	18 kV	15 cm	1.5 mL/h	20G	3.4 ± 0.011 μm

The increase in degree of molecular alignment in fibers on increasing the applied force is considered responsible for the decrease in diameter of the PLGA fibers. The web of PLGA fibers obtained at 15 kV (Fig. 4b) is found to be suitable for lamination with collagen after coating with dopamine (Fig. 4d). The architecture and pore integrity of microfibrous PLGA fabrics is found to be quite suitable for cells adhesion and proliferations.

### Coating with polydopamine

**Fig. 5:**
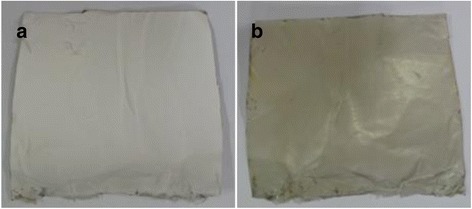
Optical image of (**a**) pristine and (**b**) polydopamine-coated PLGA

To control the surface hydrophilicity and attachment of collagen composite nanofibers, the electrospun microfibrous PLGA fabrics were immerged in 10 mM solution of Tris buffer (pH 8.5) containing 10 mg/mL dopamine. The extent of self assembled polymerization and deposition of polydopamine on surfaces of microfibrous PLGA biomaterials is found to be dependant on solution pH [36, 37] and found to sufficiently high at pH 8.5; hence, self assembled polymerization of dopamine was carried out at pH 8.5. Dopamine in alkaline medium has undergone self assembled polymerization within the pores and at the surface of microfibrous PLGA fabrics. The formation of self assembled polydopamine has produced microfibrous PLGA fabric more compact and hydrophilic due to the presence of pendant quinine along the backbone of polydopamine. The self assembled polydopamine on microfibrous PLGA fabrics has modified the surface properties of microfibers as clear from the SEM micrographs of polydopamine-coated microfibrous PLGA fabrics (Fig. 4d). In comparison to pristine microfibrous PLGA fabric (Fig. 4b), the polydopamine-coated microfibrous PLGA fabric (Fig. 1d) was having more integrated fibers than pristine PLGA fabric (Fig. 4b). The presence of pendant quinine in polydopamine is considered responsible for the attachment of biomolecules and cellular immobilization as reported in the literature [38–40]. The coating of self assembled polydopamine on microfibrous fabric has shown a color change from light brown to dark brown color. This change in color has been considered a primary indication for self assembled polymerization of dopamine via catechol oxidation to quinine, which took almost 24 h for its completion (Fig. 5). The brown color intensity is found to vary on varying the coating time for dopamine on microfibrous PLGA fabrics, which has been an indication to evaluate the extent of dopamine deposited on PLGA fabrics. No further change in color was observed on keeping microfibrous PLGA fabrics for more than 24 h; hence, it was ensured that all dopamine was consumed in formation of polydopamine on microfibrous PLGA fabrics. The spectrometric analysis of remaining solution of dopamine and washings of dopamine-coated microfibrous PLGA fabrics at 350 nm has indicated that more than 95% of dopamine was successfully consumed in the formation of self assembled polydopamine on the surface of microfibrous PLGA fabrics. The optical images of polydopamine-coated microforms PLGA fabrics were compared with pristine microfibrous PLGA fabrics (Fig. 5), which indicated for enhanced surface smoothness for microfibrous PLGA fabrics (Fig. 5b) in comparison to pristine microfibrous PLGA fabrics (Fig. 5a).

This has clearly indicated that polydopamine has not only controlled the surface functionality but also made microfibrous PLGA fabrics smoother at microscopic level. The presence of large size pores in pristine microfibrous PLGA fabrics (Fig. 4b) is found to be responsible in formation of large amount of self assembled polydopamine in the pores in comparison to formation polydopamine at the surface of microfibrous PLGA fabrics.

### Contact angle measurements

**Fig. 6:**
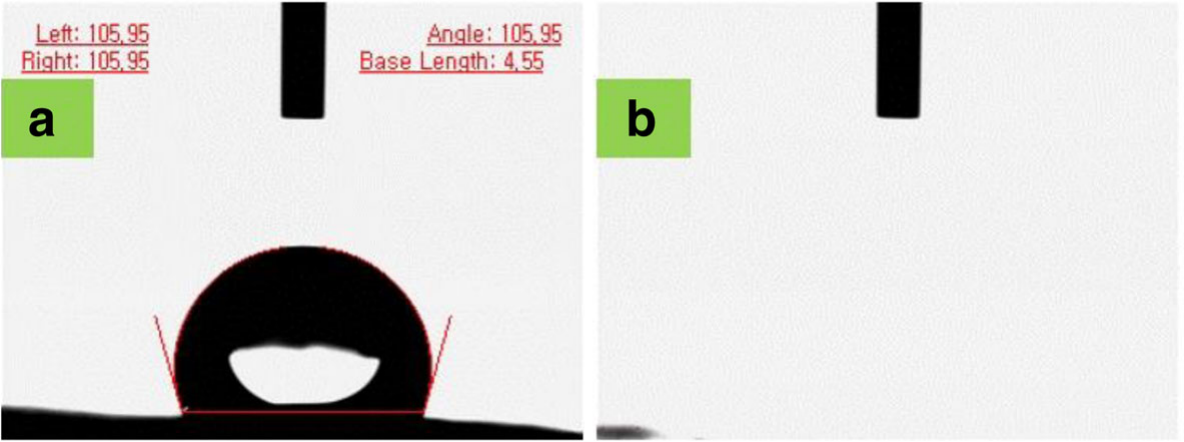
Contact angle measurements on (**a**) pristine and (**b**) polydopamine-modified microfibrous PLGA fabrics

To evaluate the variation in surface wettability of microfibrous PLGA fabrics on coating with self assembled polydopamine, the contact angle measurements of pristine microfibrous PLGA fabrics and polydopamine-coated microfibrous PLGA fabrics were carried out by sessile drop method (Kruss contact angle equipment model DS10) using deionized water, diiodomethane and formamide as reference solvents. The value of average contact angle (θ) was reported as a mean of three measurements. On comparing the average contact angles of microfibrous PLGA fabrics (Fig. 6a) and polydopamine-coated microfibrous PLGA fabrics (Fig. 6b), it is quite clear that coating of polydopamine has induced surface wettability in microfibrous PLGA fabrics in comparison to pristine microfibrous PLGA fabrics. The average contact angle (θ) has shown a significant variation from 105.9^0^ to 0^0^ (Fig. 6) on coating of polydopamine. The significant variation in the value of contact angle (θ), has provided an evidence to presume the formation of self assembled polydopamine on microfibrous PLGA through oxidation of catechol to hydrophilic quinine [38–41].

### Electrospinning of collagen-nHA composite nanofibers on microfibrous PLGA fabrics

**Fig. 7:**
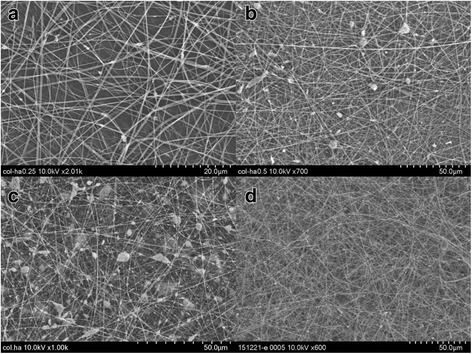
SEM micrographs of collagen nanofibers scaffolds electrospun using collagen solution containing unmodified n-HA (**a**) 3.8 wt%, (**b**) 4.4 wt%, (**c**) 5.0 wt% and (**d**) scaffolds electrospun using collagen solution containing 4.4 wt% L-glutamic acid grafted nHA

To increase the biocompatibility, cell adherence and osteogenic properties of polydopamine-coated microfibrous PLGA fabrics**,** the layers of collagen-hydroxyapatite composite nanofibers were electrospun using collagen solution with optimized amount of L-glutamic acid-grafted nHA (4.4 wt%). To enhance the cells attachment and proliferation on collagen nanofibers-laminated microfibrous PLGA fabrics, first of all collagen nanofibers scaffolds were electrospun separately using collagen solution with different amount of hydroxyapatite nanorods (Fig. 7). The amount of nHA in collagen solution was varied from 3.8-5.0 wt%, the resultant collagen nanofibrous scaffolds with unmodified nHA (Fig. 7 a, b, c) have indicated that on increasing the amount of nHA in collagen, the nHA nanorods have started the formation of aggregates instead of showing uniform distribution of nHA in the scaffolds.

**Fig. 8:**
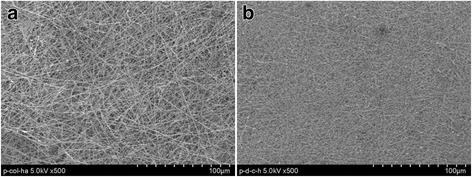
SEM micrographs of collagen-nHA composite nanofibers laminated on (**a**) pristine and (**b**) dopamine-coated microfibrous PLGA fabrics

The extent of formation of aggregates of nHA has shown an increasing trend in their size on increasing the amount of nHA from 3.8 to 5.0 wt%, which is apparent on comparing the scaffolds electrospun using collagen solution containing 4.4 wt% and 5.0 wt% of nHA (Fig. 7b, c). To produce collagen nanofibrous scaffolds having homogeneously distributed nHA, L-glutamic acid-grafted nHA was used, which produced collagen nanofibrous scaffolds having uniformly distributed nHA (Fig. 7d) at a flow rate of 1.5 mL/h and needle tip to collector distance of 15 cm and at applied voltage of 15 kV. In comparison to PLGA, the collagen was able to produce nanofibrous scaffolds under same condition of electrospinning parameters. Considering the aggregation effect of pure nHA nanorods in collagen scaffolds, the electrospinning of collagen-nHA composite nanofibers on polydopamine-coated microfibrous PLGA fabric was carried out using collagen solution containing 4.4 wt% L-glutamic acid-modified nHA. The collagen solution containing 4.4 wt% L-glutamic acid-modified nHA has produced collagen-nHA composite nanofibrous scaffolds (Fig. 7d) having homogeneously distributed hydroxyapatite nanorods (nHA-GA). After optimizing the conditions for electrospinning, the collagen-nHA composite nanofibers were electrospun on pristine microfibrous PLGA fabric (Fig. 8a) and polydopamine-coated microfibrous PLGA fabrics (Fig. 8b) till appropriate layers were deposited on PLGA fabrics, which were kept on collector. On comparing the surface morphology of pristine microfibrous PLGA and polydopamine-coated microfibrous PLGA fabrics after lamination through electrospinning of collagen-nHA-GA composite nanofibers (Figs. 5a, b), it is clear that the lamination of collagen-nHA composite nanofibers have produced smooth and compact active layer of collagen on polydopamine-coated microfibrous PLGA fabrics (Fig. 8b) than pristine microfibrous PLGA fabrics (Fig. 8a). This has clearly suggested that collagen-nHA composite nanofiber layers were having more chemical interactions with polydopamine-coated microfibrous PLGA (Fig. 8b) than with pristine microfibrous PLGA fabrics (Fig. 8a).

**Fig. 9:**
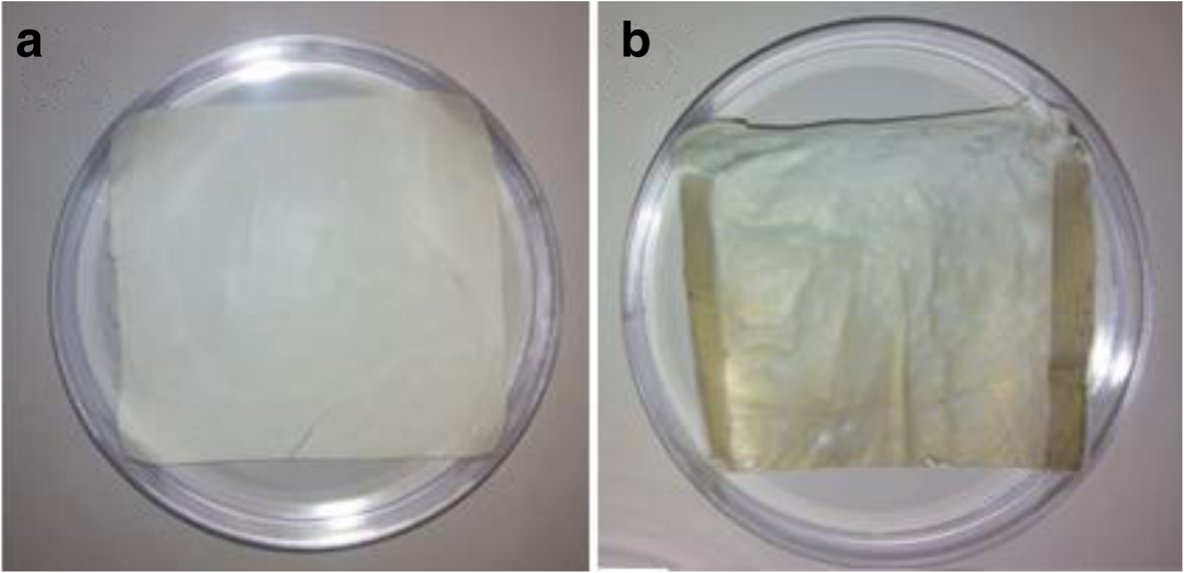
Optical images of collagen-nHA composite nanofibers laminated on (**a**) pristine and (**b**) polydopamine coated microfibrous PLGA fabrics

To compare the surface properties of pristine microfibrous PLGA and polydopamine-coated microfibrous PLGA fabrics laminated with the layers of collagen-nHA composite nanofibers, the optical microscopic images were also recoded (Fig. 9), which have suggested that the lamination of collagen-nHA composite nanofibers has contributed significantly in producing smooth and compact surfaces of microfibrous PLGA (Fig. 9b) on coating dopamine than pristine microfibrous PLGA fabrics (Fig. 9a).

**Fig. 10:**
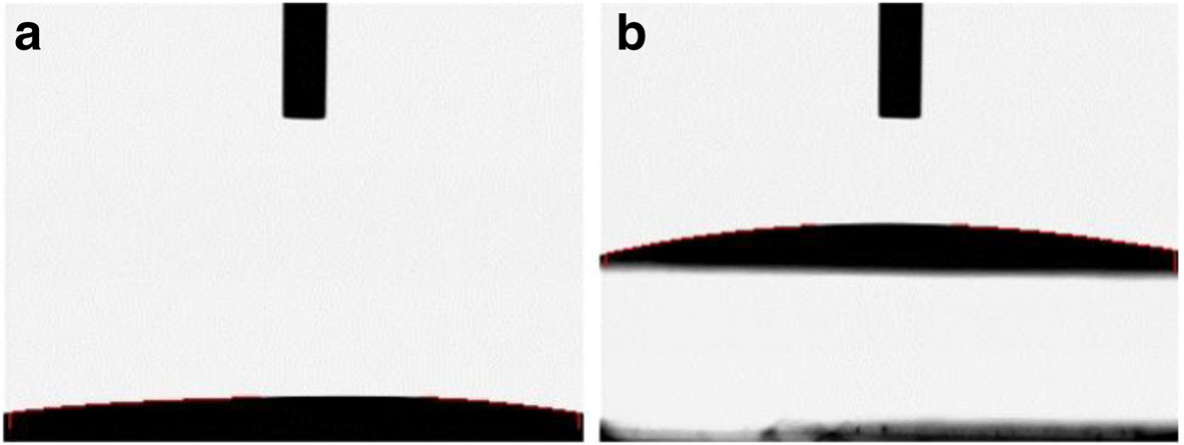
Contact angle measurements of collagen-nHA composite nanofiber laminated (**a**) pristine and (**b**) polydopamine-coated microfibrous PLGA fabrics

In addition to surface morphology, the effect of collagen-nHA composite nanofibers lamination has also been evaluated by determining surface wettability of collagen-nHA composite nanofibers-laminated pristine (a) and dopamine-coated microfibrous PLGA fabrics (b) by contact angle measurements and then value of contact angles was compared (Fig. 10).

On comparing the contact angle of pristine and polydopamine-coated microfibrous PLGA fabrics after lamination with collagen-nHA composite nanofibers, it is apparent that dopamine-coated microfibrous PLGA fabrics (Fig. 10b) produced similar wettable surfaces with pristine microfibrous PLGA fabrics (Fig. 10a). The increase in surface wettability in both cases is due to the presence of hydroxyl groups of collagen and due to the presence of n-HA nanorods at the surface of collagen-nHA composite nanofibers electrospun on pristine and dopamine-coated microfibrous PLGA fabrics, which induced surface wettability and decrease in their contact angles. These observations have clearly suggested that the lamination of PLGA fabrics has improved the surface activity to enhance cells adhesion and proliferation.

### Cells adhesion and proliferation on collagen-nHA composite nanofibers-laminated pristine and dopamine-coated microfibrous PLGA fabrics

To determine the effect of collagen-nHA composite nanofibers lamination on pristine and polydopamine-coated PLGA fabrics on adhesion of MC3T3E1 cells, the cells adhesion properties of pristine and polydopamine-coated PLGA fabrics was evaluated by seeding MC3T3E1 cells at a cell density of 3 × 10^4^ cells/cm^2^ per scaffold in a culture dish containing 500 μL non-osteogenic α-minimum essential medium supplemented with 10% fetal bovine serum, 1% penicillin/streptomycin. To compare the extent of cells adhesion on pristine PLGA and collagen-nHA composite-laminated modified PLGA, the cells were incubated for 12 h at 37 °C in presence of 5% CO_2._ After incubation, the supernatant medium was removed to Eppendorf tubes carefully and scaffolds were washed twice with phosphate buffered saline solution before fixing with aqueous solution of 2.5% glutaraldehyde for 20 min. The FE-SEM micrographs of cells-seeded scaffolds were recorded after dehydrating scaffolds in critical point drier.

**Fig. 11:**
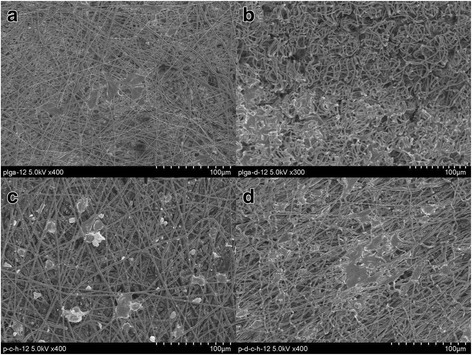
FE-SEM micrograph for studying MC3T3E1 cells adhesion behavior of (**a**) pristine, (**b**) polydopamine-coated microfibrous PLGA and of collagen-nHA composite-laminated pristine (**c**) and (**d**) polydopamine-coated microfibrous PLGA fabrics for incubation time of 12 h

On comparing the MC3T3E1 cells-seeded FE-SEM micrographs (Fig. 11), its is clear that extend of cells adhesion was lowest on microfibrous PLGA fabrics (Fig. 11a) but shown increasing trend on dopamine coating (Fig. 11b) and on further laminating with collagen-nHA composite nanofibers (Fig. 11c, d).These results have confirmed that dopamine and collagen have played a significant role in cell adhesion but in comparison to coating of dopamine, the lamination of collagen-nHA composite nanofibers has played a potential role in controlling MC3T3E1 cells adhesion (Fig. 11 c & d). Thus it is clear that dopamine has not only helped in controlling the covalent interactions with collagen but also played a synergistic effect on increasing the bioactivity of collagen; hence, the cells adhesion is found to be higher with polydopamine-coated PLGA microfibrous fabrics laminated with collagen-nHA composites nanofibers (Fig. 11d) than pristine PLGA microfibrous fabrics laminated with collagen-nHA composites nanofibers (Fig. 11c). It is also to be noticed that collagen-nHA composite nanofibers were more effective in increasing MC3T3E1 cells adhesion due to high surface area and due to presence of nHA nanorods. The significantly low cells adhesion in pristine PLGA fabric was due to the presence of microfibrous fibers in the scaffolds (Fig. 11a). The polydopamine has controlled the cells adhesion as similar to adhesion shown by muscles to all type of organic and inorganic materials through proteins secretion.

**Fig. 12:**
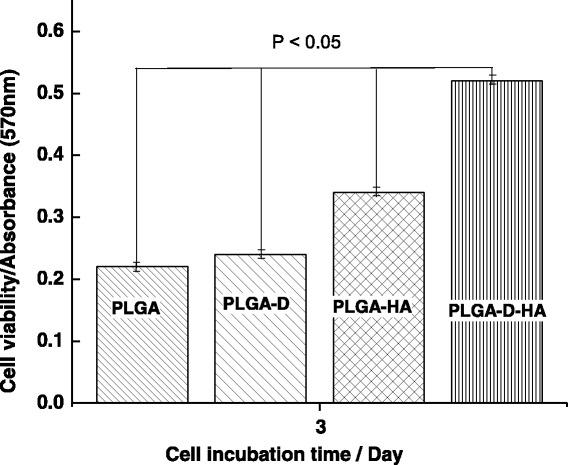
MTT assay for studying MC3T3E1 cell proliferation on (**a**) pristine, (**b**) polydopamine coated microfibrous PLGA fabrics and on collagen-nHA laminated (**c**) pristine and (**d**) polydopamine coated microfibrous PLGA fabrics for a incubation time of 3 days

The extent of MC3T3E1 cells proliferation by MTT assay was evaluated to determine the bioactivity of PLGA fabrics and PLGA fabrics laminated with collagen-nHA composite nanofibers (Fig. 12). The results of MTT assay have indicated that it is clear that the proliferation of MC3T3E1cells was more prominent with collagen-nHA composite nanofibers-laminated scaffolds (Fig. 12) and shown a significant increasing trend in cell viability from pure PLGA fabric to collagen-HA composite nanofibers laminated PLGA within a limit of standard deviation (*P* < 0.05). These trends have clearly indicated that the lamination of collagen-nHA composite nanofibers has contributed significantly to enhance the surface bioactivity for MC3T3E1 cells on using polydopamine and collagen- nHA composite nanofibers. Since the presence of polydopamine has induced the covalent interactions with collagen; hence, the activity of collagen-nHA composite nanofibers is found to be more prominent (Fig. 12) in comparison to physical interactions [42] of collagen on microfibrous PLGA fabrics (Fig. 12). The results of cells adhesion (Fig. 11) and proliferations (Fig. 12) have clearly suggested that the lamination of PLGA fabrics with collagen-nHA composite nanofibers has enhanced cells adhesion and proliferation in comparison to pristine and dopamine-modified PLGA fabrics. The L-glutamic acid-modified hydroxyapatite nanorods have played a significant role in controlling surface wetting and osteogenic properties of laminated surfaces [19–21].

## Conclusions

These studies have clearly indicated that the coating of polydopamine on microfibrous PLGA scaffolds has provided ample opportunities to modify the properties of collagen nanofibers for cells adhesion through variation in surface contact angle (θ). In comparison to PLGA, the collagen is more bioactive but its activity is possibly controlled further through covalent interactions of polydopamine at PLGA fabrics. In comparison to physical interactions, the covalent interactions of collagen in presence of self assembled polydopamine on PLGA have shown enhanced bioactivity for MC3T3E1 cells adhesion and integration through ligand-receptor interactions. The presence of L-glutamic acid-modified hydroxyapatite nanorods in collagen-composite nanofibers has also contributed toward surface wettability besides its activity in controlling the osteogenic properties of scaffolds for bone tissue engineering. The results of these studies have clearly demonstrated that collagen-nHA composites fibers may be used to create bioactive 3D scaffolds using PLGA as an architectural support agent. It is presumed that ongoing research in this area would provide more insight and information about the role of collagen-hydroxyapatite composite nanofibers in controlling the cells activities in bone tissue engineering.

## Abbreviations

ALP: Alkaline phosphatase activity
BMP: Bone morphogenetic protein
EDC: Dimethylaminopropyl ethylcarbodiimide
FDA: Food and drug association;
HA: Hydroxyapatite
nHA-GA: L-glutamic acid-grafted hydroxyapatite nanorods
NHS: N-hydroxysuccinimide
PBS: Phosphate buffer saline
PDA: Polydopamine
PEEK: Polyetheretherketone
PLGA: polylactide-co-glycolide
PLGA-Col/nHA: Microfibrous PLGA laminated with collagen-hydroxyapatite composite nanofibers
PLGA-D: Polydopamine-coated microfibrous PLGA
PLGA-D-Col/nHA: Polydopamine-coated microfibrous PLGA fabric laminated with collagen-hydroxyapatite composite nanofibers
SDS: Sodium dodecyl sulfate
SEM: Scanning electron microscope
TCP: Tricalcium phosphate
